# The Validation of the Free Fantasy Questionnaire for Children and Adolescents: From Imaginary Playmate to “Dreamtime”

**DOI:** 10.3389/fpsyg.2019.01343

**Published:** 2019-06-07

**Authors:** Renato Donfrancesco, Claudio Vezzani, Giuliana Pinto, Lucia Bigozzi, Angela Dibenedetto, Maria Grazia Melegari, Paola Gregori, Elda Andriola, Francesca Di Roma, Alessia Renzi, Renata Tambelli, Michela Di Trani

**Affiliations:** ^1^Department of Education Science, Roma Tre University, Rome, Italy; ^2^Departments of Languages, Literatures and Intercultural Studies and Education and Psychology, University of Florence, Florence, Italy; ^3^Walden Institute, Rome, Italy; ^4^Department of Developmental and Social Psychology, Sapienza University of Rome, Rome, Italy; ^5^National Health System, “La Scarpetta” Center, Rome, Italy; ^6^Department of Human Science, LUMSA University, Rome, Italy; ^7^ASL 1 Avezzano Sulmona L’Aquila, L’Aquila, Italy; ^8^Department of Dynamic and Clinical Psychology, Sapienza University of Rome, Rome, Italy

**Keywords:** fantasy, reality/unreality, children, adolescents, questionnaire

## Abstract

Fantasy in children is a precocious and important skill. In normal subjects some imaginative events, very close to hallucinations (perception-like experiences), have been found. Therefore, a better knowledge on both fantasy and the difference between imagination and the external world is needed. The aims of this study are: (a) to validate a new questionnaire for fantasy in children and adolescents; (b) to test its clinical application in ADHD children. 1.707 participants aged 8–18 years were enrolled: 1557 were recruited from a survey in six schools, whereas 150 participants were recruited in an ADHD Center. They filled out a new questionnaire, the Free Fantasy Questionnaire for Children and Adolescents, FFQ. Statistical analyses were performed to validate the FFQ and to study five parameters of fantasy. Analyses showed good properties of the FFQ as regards factor structure and reliability. Descriptive analysis showed that: 10% of the adolescents frequently have fantasy with paracosmos and 9.5% sometimes have a fantasy with imaginary relatives. Moreover, in the 64.3% of participants of primary school, in the 34.5% of lower-secondary, and in the 27.4% of upper-secondary school Perception-like experiences, involving invisible but real personages, were found. Quality of fantasy and Lack of control on imagination are correlated with a high score in the Reality/Unreality Dimension and Perception-like experiences. As regards ADHD participants, the 40% of the group showed Perception-like experiences: the 21.66% of them reported a very high score in the dimension Reality/Unreality, have some dissociative symptoms, and the 3.33% presented a clear dissociative identity disorder. All were free from psychosis or neurologic disorders. A new questionnaire to study fantasy in children and adolescents was validated. Many children and adolescents of the general population declared Perception-like experiences. These events seem to be specific, and probably normal, features of the mind; they could be better named as “Dreamtime,” whereas only in extreme conditions they could represent a risk for dissociation.

## Introduction

Imagination is developed early on to help children to process events, achieve mastery of their emotions, enrich their social understanding and develop their communication abilities (Taylor, 1999). Since imagination represents a pivotal ability in the mental functioning of typically developing children, we can speculate that a systematic study of imagination could play an important role in the understanding of the global maturation of the mind not only in normal functioning, but also in “borderline” and pathological conditions. Probably some extreme types of imagination could be close to hallucinations. We are thinking, for example, of attenuated psychotic symptoms (APS), delusional and hallucinatory phenomena in which some insight of what is real and unreal is still maintained (Yung et al., 1998).

Some authors have hypothesized that APS could be linked to a possible risk of developing psychosis in children and adolescents (Schimmelman and Schulze-Lutter, 2012; Schimmelman et al., 2013; Welsh and Tiffin, 2014). The BEARS study (Bern Epidemiological Risk Study) and the BEARS-Kid study (Schulze-Lutter et al., 2014; Schimmelmann et al., 2015) found that 9.9% of general population subjects aged 8–40 years had APS phenomena. Overall, the younger subjects had a greater amount of unusual perceptual experiences and attenuated hallucinations, with the strongest effects around age 16. However, the results of these studies have shown that perceptual APSs seem less related to functional impairment and to a real risk of psychosis than non-perceptual APS. Moreover, Aziz (2009) published a case-series of 19 participants, children and adults, from a non-psychiatric population with complex musical hallucinations. Some authors therefore suggested distinguishing between prodromal and non-prodromal hallucinations in 8- and 18-year-old subjects, finding out to what extent the subjects display a judgment of real and not real of their “visions” or “hallucinations” (Sosland and Edelsohn, 2005). The term prodromal hallucination refers to a hallucination associated with a subsequent development of a frank psychosis. In conclusion, a strong ability to distinguish imagination from reality may be important for normal maturation of the mind. So probably, in a dimensional perspective, only the extreme of a continuum representing APS, that we can also name “perception-like” phenomena, could be defined as at risk or prodromal. This global uncertainty of the data in the literature opens a large field of exploration for students of the psychological development of children and adolescents because of the obvious questions that arise: how many children and adolescents really experience confusion between reality and imagination, how many times in their lives, and what is the impact on their developing mind? The theoretical frame of this paper is that a good beginning to studying these phenomena could be to define some parameters for a correct qualitative and quantitative description and definition of the several kinds of fantasy. The five parameters of our theoretical vision are described below.

### Content of Imagination

Having imaginary companions is one of the most frequent kinds of fantasy in childhood (Svendson, 1934), and it is correlated with good development in adolescence (Seiffge-Krenke, 1997) and creativity in adulthood (Kidd et al., 2010). In particular, about the expression “good development,” Seiffge-Krenke (1997) demonstrated that adolescents with an imaginary companion do not differ from age mates without an imaginary companion with respect to the number and closeness of friends, the role-taking and perspective-taking ability.

This type of fantasy is also frequent after preschool age concerning children aged 9 and over until adolescence (Seiffge-Krenke, 1993; Pearson et al., 2001).

Taylor et al. (2004) confirmed that, in a normal school-age child population, 65% of children up to the age of 7 have an imaginary playmate and that it was not predictive of later psychopathology even in high-risk subjects (Taylor et al., 2010). Other less studied topics are: “paracosmos,” involving imaginary people living in a fantastic place (Cohen and MacKeith, 1991; Taylor, 1999), and imaginary families, a relatively common fantasy in adopted children (Horner and Rosenberg, 1991; Rosenberg and Horner, 1991). Freud himself called “familienroman” the fantasies, related to the structure of his family, in which the child or adolescent changes his ties with his parents, creating an imaginary family or a romantic life (Freud, 1908).

### The Frequency of Imaginative Events

To the best of our knowledge, many of the cited studies do not provide data about how many times certain types of fantasies (of which some examples are given later in this paper) occurs in the daily life of the participants. It is also difficult to find papers about the percentage of participants that have frequent imagined events at different ages and the degree to which these fantasies modify the social and environmental adaptation of the child and the adolescent.

### Quality of Fantasy, Control of Consciousness, and the Ability to Distinguish Reality From Unreality

The ability to imagine a situation, a person, an event that differ from one’s present reality has been related to children’s Theory of Mind, i.e., the ability to reflect on the contents of one’s own and other’s minds. Imagination is relevant to the theory of mind since it involves an unreal world that exists purely in the mind, and being able to reflect on this virtual world. To understand false belief, children must imagine that the world could be different than it is and that this different situation is what another person believes to be true about the world (Baglio and Marchetti, 2016; Pinto et al., 2016). Researchers also documented the specific contribution that engaging in fantasy worlds brings various components of children’s ability to create alternative realities and, therefore, understand others’ minds. Engaging in fantasy worlds specifically builds up children’s ability to create alternative realities, a skill that later serves them in their understanding of others’ minds (Taylor et al., 2004). Martarelli et al. (2015) demonstrated that, after statistically controlling for age, non-verbal intelligence and language skills, theory-of-mind abilities still significantly contributed to the prediction of fantasy understanding. Similarly, Dore and Lillard (2015), in a short-term longitudinal study, showed that three fantasy measures predicted improvement on theory of mind over the time period examined, but none of the fantasy measures were related to preference for mental descriptions.

According to empirical studies, the distinction between reality and fantasy develops with age (see Wellman et al., 2001 for a meta-analysis): some researchers consider that, at the age of 3 years, children are able to distinguish between reality and fantasy, and they can also identify the difference between something invisible and something imaginary – that is, they can talk about objects that have misleading appearances (Flavell, 1986). At the age of 3 and, in some cases, even at the age of 2, children know that the pretending is intentional and they realize the difference between trying to do one thing and the action of simulating the same thing (Rakoczy et al., 2004).

Some studies have demonstrated that children aged 3–5 years are well aware of the unreality of their imaginary playmates (Taylor, 1999; Carrick and Ramirez, 2012). In addition, when children 4–8 years old are questioned about the causality of imaginary improbable conditions their answers generally suggest ordinary or plausible causes (Lane et al., 2016). Recently a meta-cognitive interpretation of unreal believing was suggested: an insufficient ability to evaluate the scope and relevance of one’s knowledge leads to an overreliance on it in evaluating reality status (Woolley and Ghossany, 2013).

Other researchers believe that the confusion between the two worlds persists until mid-childhood and sustain that children cannot correctly distinguish reality from fantasy until the age of 12 (Tullos and Woolley, 2009). Many other theories about how people are able to clearly differentiate between what is real and what is unreal—or fail to do so—have been proposed (Lillard and Woolley, 2015). It has been argued, however, that none of these theories alone can explain pretense–reality confusions and that these are best explained in terms of the combined influences of cognitive availability, empirical evidence of reality, context, affect and individual differences (Bourchier and Davis, 2002). Moreover, probably not all children develop the capacity to correctly distinguish between reality and fantasy at the same speed and in the same way (Bourchier and Davis, 2000; Bigozzi et al., 2016).

Other contexts could probably also be involved in reality/unreality distinctions. In particular, the ability to distinguish one’s own thinking and/or one’s own fantasy from the external world seems more properly involved in the development of consciousness, with special attention to the definition of what we can call “Boundaries of Consciousness” as a development of one’s own identity. The development of this skill could perhaps be better studied by analyzing the conceptualisation of Johnson (1988) of what we call “hallucination.” Johnson puts the focus on the kind of imagination instead of on what is usually named “disperception.” He considers the judgment of reality to be based on two pillars: conscious control and the quality and details of imagination. So, the last three parameters appear linked. DSM 5 seems to be very close to this point of view (American Psychiatric Association [APA], 2013), suggesting that hallucination, more than a “disperception,” is a “perception-like experience” that occurs without an external stimulus, vivid and clear, and not under voluntary control. We assumed that our theoretical suggestion that a quantitative study of the five parameters was possible and that a validation of a questionnaire as a practical tool to study fantasy in this theoretical frame was also possible. Secondly, we assumed that a quantitative study of fantasy could add new information about the contents studied in the literature improving our knowledge. As the previous literature says, we could expect many children and adolescents to have perception-like phenomena but that these experiences might be non-pathological and partially explained on the basis of the parameters suggested by Johnson. On this basis we were eventually able to provide a new interpretation of non-pathological phenomena.

Bearing in mind these hypotheses, the aims of this study were:

(a) Study 1: to validate a new questionnaire as a tool for clinical and for developmental study, the Free Fantasy Questionnaire for Children and Adolescents (FFQ); to study the five parameters previously suggested: content, frequency, quality, control of consciousness and sense of reality in a large sample from the general population aged 8–18, focusing on their change over the years; to verify the presence and the frequency of a type of imagination similar to perception-like experiences in a normal population of children and adolescents.
(b) Study 2: to explore the possible role of a high-quality fantasy and low control of consciousness in generating false attribution of reality to an image (Johnson’s theory); to check the possibility that the perception-like experiences could constitute psychotic or dissociative symptoms. The choice of a control group of children and adolescents with a diagnosis for ADHD was important because they identify a pathological sub-population which presents significant comorbidity signs with dissociative identity diseases and pre-psychotic exits, and at the epidemiological level could be the easier participants to study with these characteristics.

## Study 1: The Validation of the Free Fantasy Questionnaire

### Materials and Methods

An observational study was carried out. *N* = 1707 participants were enrolled in the study; they were divided into two groups (details are reported below).

A new questionnaire was used in this study. Initially, the questionnaire consisted of 38 items relevant for the content with theoretical constructs, and it was administered to a pilot sample of 107 participants (56 children and 51 adolescents). Then, the analysis of the frequency distributions of the answers led to the elimination of 7 items, which did not result as sufficiently clear and was not able to significantly differentiate the participants from each other. Therefore, the remaining 31 items were administered to a second sample of participants, which have been involved in the validation of the latent factorial structure of the instrument.

In its final version, FFQ is a set of 31 questions with a score ranging from 0 to 5 to assess the parameter frequency: 0 = Never, 1 = Rarely, 2 = Sometimes, 3 = Quite often, 4 = Often, 5 = Very often.

The questionnaire provides information about: three typical contents of fancy (imaginary playmate, imaginary relatives, and paracosmos); quality of fantasy; control of consciousness; and meeting with personages “invisible” for others but real for the participants (see Supplementary Appendix). The last topic tests the possible confusion between reality and fantasy, bearing in mind that the concept of invisibility is well structured from 8 years (Woolley and McInnis Brown, 2015). The questionnaires were filled out in an anonymous way, but they provided information about age, class, school and social status of the participants.

#### Participants

All participants were Caucasian. Their age varied from 8 to 18 years, (age: mean 13.62, SD 3.10); gender: males 796, females 761. The sample was balanced by year of school and by gender (Primary Education: third class 100, age = 8.48 ± 0.52, 46 males; fourth class 130, age = 9.43 ± 0.51, 68 males; fifth class 126, age = 10.37 ± 0.63, 72 males; Lower-secondary Education: first class 140, age = 11.58 ± 0.60, 76 males; second class 198, age = 12.60 ± 0.75, 98 males; third class 177, age = 13.52 ± 0.63, 88 males; Upper-secondary Education: first class 129, age = 14.62 ± 0.90, 70 males; second class 152, age = 15.66 ± 0.98, 72 males; third class 118, age = 16.61 ± 0.80, 60 males; fourth class 167, age = 17.45 ± 0.74, 85 males; fifth class 130, age = 18.28 ± 1.14, 71 males).

#### Procedures

Head teachers of six schools near Rome were contacted personally to program the recruitment of participants for the study. A written consent form was sent home to the parents of all the students (*N* = 1710). Parents of 1659 children and adolescent gave informed written consent. Students who had parents’ consent to participate in the study gave personal verbal consent. Written consent was therefore obtained from all adult participants and from the parents of non-adult participants. Ethics approval was not required, according to the decision of AIFA, reported in the Official Journal of Italian Republic on March 20 2008 (general series #76, clause 10), explained in the report of the Istituto Superiore di Sanità #15/44 (2015).

Free fantasy questionnaire was administered during school hours, in the classroom, under the supervision of a psychologist and a teacher. A subsample of participants (*N* = 76; males = 38, females = 38; mean age = 11.78, *SD* = 2.85) was selected through a convenience sampling plan, prior to the initial administration for a reassessment after 2 weeks.

The questionnaires of children whose first language was not Italian were excluded from the study sample (*N* = 102), since they may not have properly understood the item of the questionnaire. The questionnaires with all answers scored 5 or answered with a zig zag strategy were considered as casually completed and excluded (*N* = 41).

#### Data Analysis

##### Analysis 1

All the statistical analyses were performed using the statistical software used were IBM SPSS Statistics 23, and MPLUS 3.0.

We performed a classical validation of the questionnaire starting from descriptive statistics and an Exploratory Factor Analysis (EFA).

A first step of data analysis involved calculating the descriptive statistics (mean, standard deviation, skewness and kurtosis coefficients) for all the items of the questionnaire. The probability distribution of the items of the questionnaire was considered normally distributed, as the skewness and kurtosis coefficients ranged between -1/+1 (Marcoulides and Hershberger, 1997).

In the second step of data analysis, several inferential analyses were carried out. To examine the contribution of various factors to false attribution of reality to an image, we employed a cross-validation procedure (Browne and Cudeck, 1993). The total sample was divided into two subgroups, matched for gender and age of the participants. On the first subgroup an EFA was carried out, and on the second subgroup a Confirmatory Factorial Analysis (CFA) was conducted to assess the validity of the factorial structure suggested by the EFA. The EFA was implemented by using the principal axis factoring method and a Promax algorithm was used to rotate the axes. Since almost all the items were not normally distributed, the MLM algorithm (Muthén and Muthén, 1998), which is a particular method of loading estimation that is robust to violations of the normality assumption, was used to compute the EFA and CFA. In particular, the MLM algorithm uses maximum likelihood parameter estimates with standard errors and a mean-adjusted chi-square test statistic that are robust to non-normality. The MLM chi-square test statistic is also referred to as the Satorra–Bentler chi-square (Muthén and Muthén, 1998).

Cronbach’s alphas were subsequently computed for the three dimensions of the EFA.

Furthermore, a CFA was carried out on the total sample to verify the factorial structure obtained from the EFA and CFA on the two subgroups. Moreover, descriptive statistics were computed for all the factorial scores by calculating the mean of all the pertinent items for each latent dimension.

A multigroup invariance analysis was carried out in order to check the stability of the factorial structure of the questionnaire in both participants under 13 years old and those older than 13. In fact, 13 years represents the age limit generally used to differentiate preadolescence from the beginning of adolescence. The invariance was verified at two levels: metric invariance and factor covariance invariance. The invariance analysis was implemented by MLM robust algorithm.

Subsequently, the test-retest reliability of the factorial structure of the questionnaire was checked by computing the correlation between the factor scores at W1 (Wave 1) and at W2 (Wave 2), 15 days later.

##### Analysis 2

After the good validation of the questionnaire, we studied the five parameters described in the Introduction. The first parameter was Frequency of some items of special theoretical importance. A qualitative study was performed dividing the 6 possible scores of frequency (0, 1, 2, 3, 4, and 5) into three groups: 0–1, 2–3, and 4–5.

The other four parameters were studied using a more tailed factorial structure with another CFA, coherently in this operation with the literature (Petchsawanga and Duchon, 2009). The parameter “content” of fantasy was studied using three dimensions (imaginary playmate, imaginary relatives and paracosmos). The three remaining parameters were studied using the following three dimensions: quality of fantasy, control of consciousness and reality/unreality. In conclusion, four of the five parameters were studied using the six-dimension structure of the second CFA. We have suggested this six-dimensions structure bearing in mind what the literature recommends about the number of items for each dimension: at least three per dimension (Hair et al., 2010).

After the CFA validation, we studied the five parameters by age. The impact of age (primary, lower-secondary and upper-secondary school) on this six-factorial structure was examined with a multivariate analysis of variance (MANOVA). For *post hoc* analysis, Bonferroni’s *post hoc* test was used if the criterion for homoscedasticity of the variances of the three subgroups was met, or Tamhane’s *post hoc* test, if it was not.

### Results

#### Analysis 1

##### Descriptive statistics

The descriptive statistics (mean, standard deviation, skewness and kurtosis coefficients) of all the 31 items of the *Free Fantasy Questionnaire* are reported below (see the text of the questionnaire in Supplementary Appendix). For all items of the questionnaire, except for item 11, the skewness and/or kurtosis coefficients showed values that were not between -1/+1, and so the scores were not distributed coherently with a Gaussian function.

##### Exploratory and confirmatory factor analyses

The two subsamples used in the EFA and the CFA were very comparable regarding age and gender variables. The subsample of participants used in the EFA had a mean age of 13.65 ± 3.03 years and consisted of 377 males and 380 females. The subsample of participants used in the CFA had a mean age of 13.58 ± 3.16 years and consisted of 386 males and 420 females. So, the two subsamples appeared to be matched both for mean age [*F*(1, 1561) = 0.19, *p* = n.s.] and for gender (χ^2^(1) = 0.61, *p* = n.s.).

The factor loadings obtained in the EFA and in the CFA are shown in Table 1 (the loadings of CFA are reported in brackets). Items 14, 26, and 27 were eliminated from the EFA because they had loadings lower than 0.30 on all three factors and consequently their communalities were also very low. Each item showed loadings higher than 0.40 on only one latent dimension after the oblique rotation both in EFA and in CFA, coherently with the simple structure criteria (Comrey and Lee, 1992; Table 1).

**Table 1:** Factor loadings resulted by EFA (out of brackets) and CFA (in brackets) on two subsamples (the first used for EFA and the second used for CFA) matched for gender and age.

	Factors		
	
Variable	Quality of fantasy	Imaginary relatives	Imaginary playmate
	**Factor loadings**		
1.	0.57 (0.52)		
2.	0.72 (0.67)		
3.	0.51 (0.58)		
4.	0.39 (0.51)		
11.	0.55 (0.60)		
13.	0.68 (0.69)		
15.	0.56 (0.63)		
16.	0.72 (0.72)		
18.	0.70 (0.71)		
20.	0.60 (0.62)		
29.	0.55 (0.61)		
31.	0.44 (0.55)		
7.		0.76 (0.81)	
8.		0.80 (0.80)	
9.		0.76 (0.80)	
10.		0.67 (0.75)	
12.		0.46 (0.50)	
5.			0.57 (0.65)
6.			0.64 (0.69)
17.			0.77 (0.74)
19.			0.63 (0.63)
21.			0.51 (0.57)
22.			0.68 (0.65)
23.			0.74 (0.79)
24.			0.76 (0.69)
25.			0.62 (0.58)
28.			0.47 (0.67)
30.			0.73 (0.75)


The results obtained by EFA and CFA showed a three-factor latent structure. The first factor, labeled as “Quality of fantasy,” measures the ability of the participants to insert their elaborate stories in a fantastic and rich of colors and particulars context. The second factor, named as “Imaginary playmate” measures the propensity of children and adolescent to imaginary a playmate, important to share daily life experiences in their fantasies. In the end, the third and last dimension, i.e., “Imaginary relatives,” includes specific fantasies in which the participants create imaginary parents and/or brothers who do not exist or act in a fake domestic environment.

The amount of variance explained by the factors was 30.30% for “Quality of fantasy,” 10.66% for “Imaginary relatives,” and 6.43% for “Imaginary playmate.” The *Root Mean Square Residual* (RMSR) for the EFA was 0.03; anything below 0.08 is generally considered a good fit.

Cronbach’s alphas were very high (“Quality of fantasy”: α = 0.87; “Imaginary relatives”: α = 0.84; “Imaginary playmate”: α = 0.90). The goodness of fit coefficients of the CFA carried out on the second sub-sample are good: Comparative Fit Index (CFI) = 0.91, Tucker-Lewis Index (TLI) = 0.91, Root Mean Square Error of Approximation (RMSEA) = 0.04, and *Standardized Root Mean Square Residual* (SRMR) = 0.06.

The results obtained by CFA carried out on the total sample revealed the same latent structure identified in the EFA on the first sub-sample and CFA on the second sub-sample. Also, in this case, all the items showed they had only one significant loading (i.e., higher than 0.40) in only one of the three latent dimensions. The coefficients of goodness of fit of the CFA model resulted very good: CFI = 0.92, TLI = 0.91, RMSEA = 0.04, SRMR = 0.05. All three factors showed they were significantly associated by positive correlations (“Quality of fantasy” with “Imaginary relatives”: *r* = 0.33, *p* < 0.001; “Quality of fantasy” with “Imaginary playmate”: *r* = 0.56, *p* < 0.001; “Imaginary relatives” with “Imaginary playmate”: *r* = 0.57, *p* < 0.001).

In general, the results pointed out by EFA and CFA showed the statistical validity of the factorial structure obtained, and allows to consider the three factorial dimensions as three real and consistent psychological constructs.

The descriptive statistics regarding the three factorial dimensions, carried out for each level of school grade (primary school, lower-secondary school and upper-secondary school), are reported in Table 2. The factor scores were calculated by computing (on the total sample) the mean of the items of each latent dimension.

**Table 2:** Descriptive statistics of the three-factor scores of the *Free Fantasy Questionnaire*.

Scholastic class	Variable	Min	Max	M	SD	Sk	Ku.
Primary school	Quality of fantasy	0	5	2.10	1.18	0.249	-0.533
(*N* = 356)	Imaginary relatives	0	5	0.69	1.01	1.66	2.23
	Imaginary playmate	0	5	1.20	1.13	0.89	0.03
Lower-secondary school	Quality of fantasy	0	5	1.55	1.09	0.519	-0.455
(*N* = 516)	Imaginary relatives	0	5	0.45	0.85	2.63	7.59
	Imaginary playmate	0	5	0.51	0.80	1.97	3.50
Upper-secondary school	Quality of fantasy	0	5	1.55	1.06	0.620	-0.160
(*N* = 691)	Imaginary relatives	0	5	0.39	0.74	2.62	7.92
	Imaginary playmate	0	5	0.35	0.60	2.42	5.85


###### Multigroup model

Regarding the multigroup analysis used to check the invariance of the factorial structure of the questionnaire between the participants less than or more than 13 years old, the results confirmed the metric invariance of the factorial structure of the questionnaire in the two subgroups (less vs. more than 13 years old; CFI = 0.88, RMSEA = 0.06, SRMR = 0.07) (Meredith, 1993; Steenkamp and Baumgartner, 1998). The questionnaire thus appears to measure the same three latent dimensions in the two age subgroups, with the same factorial loadings.

The results of the test-retest indicated good reliability of the latent structure, for “Quality of fantasy” (*r* = 0.73), for “Imaginary relatives” (*r* = 0.76) and for “Imaginary playmate” (*r* = 0.76). The three factorial dimensions seem to show significant consistency between W1 and W2.

#### Analysis 2

##### Frequency distributions of some very theoretically relevant items

The study of the five parameters (frequency, content, quality, control of consciousness and Reality/Unreality) begins with the results for the qualitative analysis of the frequency distribution of the items. In Table 3 the percentage of distributions of the answers to specific items in the primary, lower-secondary and upper-secondary grades regarding an imaginary playmate created by participants is shown. Table 3 also shows the distribution of items regarding a fictional environment. As can be seen, the frequency of the answers “2–3” and “4–5” is very high for items 5 and 6, regarding the presence of an imaginary playmate, in particular in primary school but also in lower- and upper-secondary school. The same result was obtained for items 15, 26, and 27, regarding a fictional environment (Table 3). Also shown in Table 3 is the frequency distribution of some particular theoretically important items, regarding the domain that we could call “Reality/Unreality,” in which the participants asserted the “reality” of their own fantasy representations, such as the real existence of an imaginary playmate, or regarding a fantastic world (Table 3). These items were selected because they were more theoretically relevant for the specific construct measured, coherently with the specialized scientific literature.

**Table 3:** Percentage of distributions of the answers to specific items regarding the imaginary playmate, imaginary relatives, paracosmos and the ability to differentiate the reality to unreality.

School	Factor	Variable	Scores 0–1	Scores 2–3	Scores 4–5
Primary school	Imaginary playmate	Item 5	59.3	21.1	19.7
		Item 6	64.6	17.7	17.7
	Imaginary relatives	Item 9	84.0	11.2	4.8
	Paracosmos	Item 15	54.2	21.6	24.2
		Item 26	55.1	27.0	18.0
		Item 27	51.7	24.7	23.6
	Reality/Unreality	Item 17	68.5	18.3	13.2
		Item 19	69.1	19.1	11.8
		Item 22	75.6	13.5	11.0
		Item 23	66.9	19.1	14.0
		Item 24	72.5	16.6	11.0
		Item 25	79.2	11.2	9.6
		Item 28	63.2	20.8	16.0
Lower-secondary school	Imaginary playmate	Item 5	83.3	10.4	6.3
		Item 6	84.0	9.7	6.2
	Imaginary relatives	Item 9	90.2	6.5	3.3
	Paracosmos	Item 15	72.3	15.5	12.2
		Item 26	76.7	13.1	10.2
		Item 27	75.7	13.2	11.0
	Reality/Unreality	Item 17	86.9	7.0	6.1
		Item 19	85.3	10.2	4.5
		Item 22	91.0	5.1	3.9
		Item 23	88.5	7.6	3.9
		Item 24	89.4	7.1	3.5
		Item 25	93.2	3.3	3.5
		Item 28	81.7	13.0	5.3
Upper-secondary school	Imaginary playmate	Item 5	87.4	8.5	4.1
		Item 6	91.0	5.6	3.4
	Imaginary relatives	Item 9	90.5	7.6	1.9
	Paracosmos	Item 15	72.2	17.9	10.0
		Item 26	78.0	14.2	7.8
		Item 27	83.8	11.2	5.0
	Reality/Unreality	Item 17	90.9	6.0	3.1
		Item 19	89.9	6.7	3.4
		Item 22	94.6	4.4	1.0
		Item 23	92.5	6.1	1.3
		Item 24	93.9	4.8	1.3
		Item 25	94.3	3.8	1.9
		Item 28	85.8	9.5	4.7


*Item 5 and 6, Imaginary playmate; Item 9, Imaginary relatives; Item 15, 26, and 27, Paracosmos; Item 17, 19, 22, 23, 24, 25, 28, Reality/unreality. Scores: 0 = Never; 1 = Rarely; 2 = Sometimes; 3 = Quite often; 4 = Often; 5 = Very Often.*

In general, the frequency distribution of these items appears very similar at different ages. For primary school students, the answers “2–3” are ranged between 11.2 and 24.7%, and this percentage remains very high in lower-secondary and upper-secondary school, though significantly lower. Finally, the percentage of answers with score 2 (Sometimes) in at least one item of the items 17, 19, 22, 23, 24, 25, 28, and 30, concerning playing or meeting with an invisible (for others) but real personage is surprisingly high: 64.3% in primary school, 34.5% in lower-secondary and 27.4% in upper-secondary school.

##### CFA of the six-factor latent structure and multigroup CFA model

After frequency the other four parameters were studied by using a structure with six dimensions: the first three dimensions concern the parameter “Content” (Imaginary Playmate, Imaginary relatives and Paracosmos). The other three dimensions concern the parameters Quality of fantasy, Control of Consciousness and Reality/Unreality judgement.

In addition to the three factorial structure pointed out by the first factorial solution, this six-factor latent structure introduced three further dimensions.

High scores in the “Paracosmos” factor describe a child/adolescent who, in his/her fantasies, enter in a world very different from the of everyday life world and is conceptualized as created by subject. In this fantasy world, the rules are very different respect to the roles of the everyday life world, where (for example) the child/adolescent is invisible.

High scores in the “Control of Consciousness” factor describe a child/adolescent who is not sure if his/her fantasies were something real of a dream, fantasies such as these seem to have entered in his/her head from another person and over which he/she doesn’t have control.

High scores in the “Reality/Unreality” factor describe a child/adolescent who think that a fantasy and invisible character can really exist and only he/she can call by doing something, or he/she can ask for help to change something he/she doesn’t like, or that advises him/her on what to say and do.

It was carried out a CFA with the aim to check the validity of a similar latent structure, alternative to the three-factor solution. The result of the CFA regarding the six-factor latent structure showed very good construct validity. The goodness-of-fit coefficients are good: CFI = 0.91, TLI = 0.90, RMSEA = 0.04, and SRMR = 0.08. The loadings for the CFA implemented on the total sample are reported in Table 4.

**Table 4:** Factor loadings resulted by CFA on the six-factor latent structure in the total sample.

	Factors					
	
Variable	Quality of Fantasy	Control of the consciousness	Imaginary playmate	Imaginary relatives	Paracosmos	Reality/Unreality
**Factor loadings**						
1.	0.51					
2.	0.66					
13.	0.68					
16.	0.72					
18.	0.72					
29.	0.61					
4.		0.48				
21.		0.58				
30.		0.68				
5.			0.77			
6.			0.81			
31.			0.40			
7.				0.80		
8.				0.82		
9.				0.76		
10.				0.75		
15.					0.68	
26.					0.72	
27.					0.73	
17.						0.71
19.						0.65
22.						0.69
23.						0.79
24.						0.72
25.						0.61
28.						0.75


Therefore, in the conclusion it is possible to consider two alternative factorial solutions for the questionnaire: one with three latent dimensions and a second with six latent factorial dimensions.

The descriptive statistics for the six-factorial solution for each school grade (primary school, lower- and upper-secondary school) are reported in Table 5. The factor scores were calculated by computing the mean of the items in each latent dimension, that is, in the same way that was used with the three-factor solution.

**Table 5:** Descriptive statistics about the six-factor solution of the *Free Fantasy Questionnaire* for each school class.

School	Variable	Min	Max	M	SD
Primary school	Imaginary playmate	0	5	1.85	1.45
(*N* = 356)	Imaginary relatives	0	5	0.61	1.03
	Paracosmos	0	5	1.76	1.44
	Quality of fantasy	0	5	2.16	1.31
	Control of consciousness	0	5	1.42	1.26
	Reality/Unreality	0	5	1.10	1.16
Lower-secondary school	Imaginary playmate	0	5	1.01	1.13
(*N* = 516)	Imaginary relatives	0	5	0.40	0.84
	Paracosmos	0	5	1.00	1.25
	Quality of fantasy	0	5	1.62	1.21
	Control of consciousness	0	5	0.86	0.99
	Reality/Unreality	0	5	0.44	0.80
Upper-secondary school	Imaginary playmate	0	5	0.78	0.95
(*N* = 691)	Imaginary relatives	0	5	0.32	0.75
	Paracosmos	0	5	0.83	1.09
	Quality of fantasy	0	5	1.67	1.19
	Control of consciousness	0	5	0.77	0.88
	Reality/Unreality	0	5	0.28	0.60


The “Reality/Unreality” dimension was significantly correlated with the “Quality of fantasy” (*r* = 0.46, *p* < 0.001) and “Control of consciousness” (*r* = 0.89, *p* < 0.001) dimensions. This result shows that these three dimensions were resulted associated by a directly proportional relationship.

##### Mean differences in the fantasy dimensions between different ages

The results obtained by MANOVA, used to predict the statistical impact of the participants’ grade (primary, lower- and upper-secondary school) on six factorial dimensions of the questionnaire, showed the significance of the overall model (Grade: Wilks’ Lambda = 0.826, *F*(12, 3112) = 26.05, *p* < 0.001). In particular, the *post hoc* test of Bonferroni (or Tamhane’s *post hoc* test) showed that the mean score of participants attending primary school was higher than that for lower-secondary school students (Imaginary playmate: Tamhane = 10.68, *p* < 0.001, lower C.I. 0.29, upper C.I. 0.45; Imaginary relatives: Tamhane = 5.58, p < 0.001, lower C.I. 0.29, upper C.I. 0.72; Paracosmos: Tamhane = 9.76, *p* < 0.001, lower C.I. 0.24, upper C.I. 0.40; Quality of fantasy: Tamhane = 7.27, *p* < 0.001, lower C.I. 0.29, upper C.I. 0.57; Control of consciousness: Tamhane = 10.36, *p* < 0.001, lower C.I. 0.19, upper C.I. 0.30; Reality/Unreality: Tamhane = 10.87, *p* < 0.001, lower C.I. 0.41, upper C.I. 0.64) and upper-secondary school students (Imaginary playmate: Tamhane = 14.58, *p* < 0.001, lower C.I. 0.38, upper C.I. 0.54; Imaginary relatives: Tamhane = 8.26, *p* < 0.001, lower C.I. 0.50, upper C.I. 0.90; Paracosmos: Tamhane = 12.08, *p* < 0.001, lower C.I. 0.29, upper C.I. 0.43; Quality of fantasy: Tamhane = 7.74, *p* < 0.001, lower C.I. 0.29, upper C.I. 0.56; Control of consciousness: Tamhane = 13.33, *p* < 0.001, lower C.I. 0.24, upper C.I. 0.34; Reality/Unreality: Tamhane = 15.02, *p* < 0.001, lower C.I. 0.56, upper C.I. 0.77) for all six factorial dimensions.

The same significant results were obtained regarding the difference between lower- and upper-secondary school participants for “Imaginary playmate,” “Imaginary relatives,” and “Reality/Unreality” (the scores were higher for middle than for upper-secondary school children), while no differences were identified for “Paracosmos,” “Quality of fantasy,” or “Control of consciousness,” in which the mean scores had the same value (Imaginary playmate: Tamhane = 3.74, *p* < 0.001, lower C.I. 0.03, upper C.I. 0.15; Imaginary relatives: Tamhane = 2.72, *p* < 0.05, lower C.I. 0.02, upper C.I. 0.37; Paracosmos: Tamhane = 1.67, *p* < n.s., lower C.I. -0.02, upper C.I. 0.10; Quality of fantasy: Tamhane = 0.03, *p* = n.s., lower C.I. -0.11, upper C.I. 0.11; Control of consciousness: Tamhane = 2.65, *p* = n.s., lower C.I. -0.01, upper C.I. 0.08; Reality/Unreality: Tamhane = 4.14, *p* < 0.001, lower C.I. 0.06, upper C.I. 0.22).

### Discussion

#### Analysis 1

Our primary hypothesis about the possibility of producing a questionnaire as a tool to study the phenomenon of fantasy in children and adolescents was supported. Indeed, in the first part of the results section we reported that the “Free Fantasy Questionnaire” is a good and validated tool to study fancy in children and adolescents. To our knowledge, FFQ is the first validated questionnaire to study fantasy in a large age range (between 8 and 18 years). In addition, this questionnaire addresses all main issues of fantasy as reported by literature (imaginary playmate, etc …). Moreover, also the first of our secondary hypotheses, i.e., that a quantitative study of fantasy could add new information about the contents studied in the literature improving our knowledge, it is supported by the results because new information is now added about our knowledge of fantasy in children and adolescents.

The statistical analyses in the exploratory section, in the Cronbach Alpha study, and in the confirmatory section (analysis performed with the half of the sample that was not used for EFA and successively the CFA for the total sample), the study of invariance (homogeneity through the ages) with a multigroup model analysis, and the study of the stability of the questionnaire (Test-Retest) all supported its reliability and validity for all the ages explored. The factor loadings obtained with EFA (on a first stratified split half sub-sample) and CFA (on a second stratified split half sub- for sample) showed a factorial structure with three dimensions: Imaginary playmate, Imaginary relatives, and Quality of fantasy. Table 2 reports mean and SD for these three dimensions for children and adolescents of the survey at different ages. All three of these dimensions provide a great number of interesting answers in all the ages tested, and probably they give a good description of what is a large part of a daydream from age 8 to 18. The dimension “quality of fantasy” measures with several items the ability of the participants to invent long and elaborate stories, how much the child or the adolescent is prone to insert her/his story in a context such as a fantastic city, and how rich the fantasy is in particulars and in vivid colors. This dimension explains 30% of the variance. Fantasizing about an imaginary playmate is a second dimension of the questionnaire, with an explained variance of 6.43%. The items concern the play of the children and adolescents with an imaginary playmate. The personage could be invented by the participants with full awareness of its pretend quality or, on the contrary, it could be considered “real” but visible only to the subject. As shown in Table 2, participants of all ages reported having an imaginary playmate, although this became less frequent with age. Finally, the FFQ also documents the presence of daydreams about imaginary families in a large normal non-adoptive population, with an explained variance of 10.66%. The items of this dimension concern the presence or not of a fantasy about different relatives, house or parents from the real one of the child or the adolescent. These last results are a novelty in empirical studies. Indeed, the presence of this specific dimension of fantasy was only speculated in some papers on the basis of anecdotal reports (Horner and Rosenberg, 1991; Rosenberg and Horner, 1991).

Similarities and differences between our results and the previous literature will be addressed in the discussion of analysis 2.

The composition of the sample may be a limitation to generalizability of the results of this analysis. The sample was recruited only in the central part of Italy and may not represent the overall Italian population because the localized geographical distribution of the participants. In addition, this tool would need a specific validation to be introduced in other countries.

#### Analysis 2

Our results supported the secondary hypothesis that it is possible to study Fantasy using the 5 parameters presented in the Introduction.

The first part of Analysis 2 consists of a qualitative study of the parameter “Frequency” of some items as reported in Table 3. Our results about the frequency of imaginary playmate through the ages confirm the results of previous research in pre-adolescence and adolescence (Seiffge-Krenke, 1993; Pearson et al., 2001; Taylor et al., 2004). Nevertheless, imaginary playmate becomes an infrequent fantasy by upper-secondary school age.

A novel aspect of the FFQ is the introduction of a number of items regarding a “Real Personage” invisible for other people but visible for the subjects. The qualitative study of the parameter “Frequency” in this section is very interesting but not “astonishing” as some might suggest at first glance. These items are formulated in such a way that misunderstanding is difficult to occur. Moreover, it is not likely that answers to these kinds of items are based on a simple disperception, because the items are centered on a character that comes to mind or that presents himself or herself without collaboration of the subject. This character may be located in a complex “Paracosmos” (Item 30), may be an alien, may order the subject to do something, or may give suggestions for the solution to problems (see Supplementary Appendix). It is not a simple hallucination but a hallucination with a sort of unspecified and lucid “delirium.” Indeed, the percentage of answers with score 2 (Sometimes) in at least one of these types of items is surprisingly high: 64.3% in primary school, 34.5% in lower-secondary school and 27.4% in upper-secondary school. If we compare these results with those of previous studies about APS, we can see a difference. As was said in the introduction, the BEAR study and the BEAR-Kid study (Schimmelmann et al., 2015) found that 9.9% of a group of participants aged 8–40 representative of the general population have APS phenomena. This percentage is greater but not far from our data, if we consider the percentage of answers with score 4 or 5, Often and Very often (Table 3).

The biggest difference from previous study results is due to answers with score 2 or 3, Sometimes or Sometimes yes sometimes no. The difference between our results and those of the BEAR and BEAR-Kid study might be due to the inclusion of some phenomena with a less frequent manifestation (sometimes) in the present research. Moreover, the difference might be due to the different methodology: questionnaire versus interview. In any case, our results suggest the possibility of a latent unitary structure with a specific dimension that we can name Reality/Unreality (see below). The trend to daydream with a sort of “Paracosmos” interests, following the literature, the late primary school and the pre-adolescence (Taylor, 1999). Our results are consistent with these data. Item 15 describes a basic level of Paracosmos in which the child or the adolescent enters an imaginary world. As expected, children of primary school age are well represented and 24.2% of them answer with score 4 and 5 (see Table 3). A novel finding is that the adolescent population also has this type of daydream, with 10% of answers with score 4 or 5, Often or Very often.

Children of primary school age answer “Sometimes” or greater (from 2 to 5) 15% of the time for item 9 that asks if they fantasize about Imaginary relatives (item 9). This percentage decreases to 9.5% for upper-secondary school participants. To our knowledge this is the first study that documents this type of daydream in the general population, suggesting the idea that play with imaginary parents is not exclusive to adopted children.

As suggested by the literature, a good validation of the questionnaire permitted us to perform a second analysis with a new CFA (Petchsawanga and Duchon, 2009). This was based on a new functional structure following what the literature suggests.

The structure studied with a new CFA has 6 factors describing the four parameters that we still have to study (see Table 4): three factors concern the parameter “content,” 1- Imaginary playmate (but only when the child is aware of being the subject that creates the daydream), 2- Imaginary relatives, 3- Paracosmos. The other three dimensions concern the other three parameters: 4- Quality of fantasy, 5- Control of consciousness, and 6- Ability to distinguish reality/unreality. This last dimension is formed by the 7 items describing a meeting with an invisible but real character (Items: 17, 19, 22, 23, 24, 25, and 28).

We found that the CFA with six dimensions had a very good result (see Table 4). This confirms the latent structure of the dimension concerning reality and unreality of imagination. Table 5 provides means and SDs for all six dimensions and all ages. These results cannot be compared with previous research. Indeed, to our knowledge a theoretical framework based on a quantitative study of some parameters suggested by the literature has been proposed for the first time. This quantitative evaluation may allow future researchers to assess these parameters and could probably provide a quantitative definition of “adolescent at risk.”

We found a large “age effect.” Table 6 shows the results of MANOVA and *post hoc* analysis. As we have said above, this analysis enables us to study the quantitative profile of the frequencies by comparing the score of every dimension for each age. There are two different kinds of dimensional development (bearing in mind that these data are cross-sectional and not longitudinal): the Imaginary playmate, Imaginary relatives and Reality/Unreality dimensions have a progressive and gradual reduction of the score through the ages. Probably the significant difference not only between primary school and lower-secondary school but also between lower- and upper-secondary school indicates that in upper-secondary school this topic is present in the fantasy of adolescents but also that it is not frequent, confirming what we have seen in the qualitative study of the frequency. The same trajectory is followed by the score of the items concerning the Imaginary relatives, a topic generally attributed to adopted children (Horner and Rosenberg, 1991; Rosenberg and Horner, 1991).

**Table 6:** Differences (MANOVAs) between the three schools observed on the six-factor scores.

Independent variable	Dependent variable	*SS*	*df*	*MS*	*F*	*p*	*Post hoc*	*η^2^*
Schools	Imaginary playmate (^∗^)	51.23	2	25.62	132.07	<0.001	1<2, 1<3	0.145
	Imaginary relatives (^∗^)	114.85	2	57.43	36.75	<0.001	1<2, 1<3,	0.045
	Paracosmos (^∗^)	33.11	2	16.55	83.66	<0.001	1<2, 1<3, 2<3	0.097
	Quality of fantasy	49.84	2	24.92	38.25	<0.001	1<2, 1<3, 2<3	0.047
	Control of the consciousness (^∗^)	20.66	2	10.33	112.98	<0.001	1<2, 1<3	0.127
	Reality/Unreality (^∗^)	105.88	2	52.94	139.90	<0.001	1<2, 1<3, 2<3	0.152
Error	Imaginary playmate (^∗^)	302.58	1560	0.19				
	Imaginary relatives (^∗^)	2437.33	1560	1.56				
	Paracosmos (^∗^)	308.68	1560	0.20				
	Quality of fantasy	1016.45	1560	0.65				
	Control of the consciousness (^∗^)	142.66	1560	0.09				
	Reality/Unreality (^∗^)	590.31	1560	0.38				


*Imaginary playmate, *R*^*2*^ = 0.15 [*F*(2, 1560) = 132.07, *p* < 0.001]; Imaginary relatives, *R*^*2*^ = 0.05 [*F*(2, 1560) = 36.76, *p* < 0.001]; Paracosmos, *R*^*2*^ = 0.10 [*F*(2, 1560) = 83.66, *p* < 0.001]; Quality of fantasy, *R*^*2*^ = 0.05 [*F*(2, 1560) = 38.25, *p* < 0.001]; Control of consciousness, *R*^*2*^ = 0.13 [*F*(2, 1560) = 112.98, *p* < 0.001]; Reality/Unreality, *R*^*2*^ = 0.15 [*F*(2, 1560) = 139.91, *p* < 0.001]. *Post hoc* tests: 1, primary school; 2, lower-secondary school; 3, upper-secondary school. (^∗^), factor score was normalized by increasing monotonic transformations.*

To our knowledge, this is the first paper with a large community-based survey about “Imaginary relatives.” As we have seen, the topic is present in the community-based sample, but far more frequently among primary school age children. After this age, daydreams about Imaginary relatives decrease progressively. There is also a progressive decrease in the frequency of the reality/unreality dimension from primary to lower-secondary school and from lower-secondary to upper-secondary school. According to our interpretation, the progressive lowering of scores in that dimension suggests that the development of the boundaries of consciousness is slow, gradual, and, for some, not yet finished by adolescence. We defined the “boundaries” of consciousness as “The ability to distinguish external reality from her/himself.” More precisely, this ability consists of two aspects: the first is characterized by the ability to attribute a subject to thoughts, and to attribute the property to oneself. The second is to understand that thought and sensitive perception correspond to non-commensurable categories. This process, in our opinion, contributes to the formation of identity.

This developmental process is probably part of a more general development of consciousness, especially in the process of definition of identity as a complex and articulated cluster of boundaries between the mind of the subject and the external world. But it is also probably true that this development is not linear for all children (Woolley, 1997). For some this maturation could be relatively rapid (Taylor, 1999: Carrick and Ramirez, 2012). The dimension “Control of Consciousness” has a significantly lower average score for lower-secondary school than primary school students, indicating, according to our interpretation, a major advance in conscious control. In the same way a decreasing score in “quality of fantasy” was found between primary and lower-secondary but not between lower-secondary and upper-secondary school students. Control of consciousness and quality of fantasy are the two variables suggested by Johnson (1988) to define hallucination, and both are strongly correlated to the dimension Reality/Unreality. These results indirectly confirm the hypothesis of Johnson because they show that a perception-like phenomenon is more likely to happen the more a subject is prone to low conscious control and to high quality of fantasy. Obviously, the limitation of these results is due to the fact that we did not study the perception-like phenomenon in the moment of its production by the subject. Our data only suggest that this phenomenon is more likely to happen in participants who are prone to have a lower control of consciousness and a better quality of fantasy in comparison with the general population. Finally, Table 6 also shows that “Paracosmos” fantasies are typical of late primary school age children, although it may be present in adolescents, with a strong decrease from primary to lower-secondary school but similarity between lower-secondary and upper-secondary school scores. According to our interpretation, the decrease of frequency of this type of fantasy with growing age, may be due to this issue: we found a general decrease of quality and quantity of fantasy from childhood to late adolescence.

This study has some limitations. First, the data of the survey are not all longitudinal, and this is a limitation in a study of developmental psychology. The children and adolescents surveyed who gave positive answers to the items of the “Dreamtime” dimension could be interviewed in person to better verify any difference between several kinds of imagination and perception-like phenomena. Furthermore, a sample with a better accurate consideration of the effects due to the socio-economic differences among the participants would have provided more consistent results. Finally, another limit of the present study is that, in the data collected, there is no information about use of computers, tablets and, in general, the familiarity with virtual reality, to be compared with the familiarity with more common forms of narratives. Future studies will also need to investigate and take under control these aspects within the design of the research.

## Study 2: First Applications of the FFQ in a Clinical Sample

The research was performed in Italy where it is very difficult to have the parents’ permission to visit their children if normal and during a scholastic survey, using a psychiatric interview and electroencephalography (EEG). The clinical assessment for ADHD on the contrary is requested by the same parents and it gives us the opportunity to study the dimensional impact on FFQ of any possible perception-like phenomenon and its possible categorial expression. DSM 5 indeed requests (for ADHD) a differential diagnosis between ADHD and possible psychosis or dissociative identity disorder focusing on the conditions in which the boundaries of consciousness are weak, which is what we were seeking. We remember that dissociative identity disorder, differently from psychosis, is a complex anxiety disorder in which the subject shows for a variable period of time a behavior and a perception of her/himself corresponding to several apparent personalities; sometimes the subject has the feeling that someone has entered her/himself.

### Materials and Methods

#### Participants

Children (*N* = 150) were recruited as consecutive outpatients referred to an ADHD center for diagnosis, from June 1 2014 to December 31 2015 (age: mean 11.07, SD 2.30; gender: males 125, females 25). This ADHD center was located in the same area in which the participants were recruited in the schools. All participants were Italian speakers as a first language. All were Caucasian. All participants had an IQ >70 on the WISC III Scale (Wechsler, 1991), because cases are selected before referral to the ADHD center. All parents provided written consent to the research and all the 150 participants provided verbal consent. All 150 participants were totally naive about any kind of drug. We point out that the Italian regulation states that methylphenidate may be prescribed only following the decision of an ADHD center. The use of psychedelic drugs by the adolescents was excluded by an anamnestic interview to the parents and the participants.

#### Procedures

The FFQ was completed in a quiet room and contained also information about age, school year and social class. The participants were sampled though a convenience sampling plan.

All 150 children and adolescents visited in the ADHD center filled out the FFQ questionnaire. K SADS PL 1.0, a semi-structured psychiatric interview, well known and used worldwide (Kaufman et al., 1997) was performed by only one and expert child neuropsychiatrist (R.D.). This standardized tool enables the interviewer to diagnose ADHD and the most frequent psychiatric disorders in children and adolescents. K-SADS is also useful to evaluate any psychotic or schizophrenic condition. The participants were also studied by a psychiatric evaluation and anamnesis. All these children also underwent an EEG and a neurological examination to exclude any form of epilepsy, tumor, or neurological condition that could be responsible for a perception-like experience.

#### Data Analysis

Descriptive statistics were calculated for the data from the 150 children and adolescents from the ADHD center. A Fisher exact test was performed to compare groups with different frequencies of symptomatology with special attention to dissociative symptoms.

### Results

There were 147 children and adolescents in the clinic sample who were diagnosed with ADHD, two were diagnosed with ADHD with a comorbidity of a superimposed Dissociative Identity Disorder, and one was diagnosed as having only a Dissociative Identity Disorder alone without ADHD. Sixty (40%) answered at least one item of the Reality/Unreality questions with a score 2 (sometimes). They all had a negative profile for epilepsy or other major neurological disorders, for psychosis and schizophrenia.

Thirteen participants of the 60 with a score of 2 on the dimension Reality/Unreality (21.66% of this group of participants and 8.66% of the total center group) exhibited “dissociative symptomatology,” defined as the presence of criteria A or B of the DSM 5 Dissociative Identity Disorder Criteria without full agreement, on the psychiatric assessment. For example, the symptom may be present in an intermittent or occasional way or without a clear impact on the life of the child or adolescent (Criterion C). In all these participants a “fantasy” about an invisible character was part of this incomplete symptomatology. In other words, in these participants the personage “entered” themselves occasionally or sporadically with an occasional or absent impact on their behavior. This is a typical dissociative symptom according DSM 5 criteria. There were two females and 11 males; the mean age was 11.63 months. The mean score in the dimension Reality/Unreality of these 13 participants was 17.0. This is a very high score: +20.7 SD for this dimension in lower-secondary (paying attention to the mean age of these children). Two adolescents in this group developed a clear Dissociative Identity Disorder diagnosed according to DSM 5 Criteria. They were 3.33% of the group with a significant score on the Reality/Unreality dimension and 1.33% of the total center group. Of the 90 participants without items with score 2 in the dimension reality/unreality, only two were diagnosed as having dissociative symptomatology and none were diagnosed with Dissociative Identity Disorder. On the Fisher Exact Test, the difference between the two groups with 60 and 90 participants, respectively regarding the number of participants with a dissociative symptomatology (13 vs. 2) was significant; (Fisher Exact Test: *p* < 0.001; Cramer *V* = 0.32, *p* < 0.001). In particular, significantly more cases of dissociative symptomatology were observed in participants with at least one item with a score of 2 (*Frequency observed* = 13; *Frequency attended* = 6; *Adjusted standardized residual* = 3.9) and less cases in the participants without a score of 2 (*Frequency observed* = 2; *Frequency attended* = 9; *Adjusted standardized residual* = -3.9), whereas more cases of absence of dissociative symptoms were observed in participants without a score of 2 (*Frequency observed* = 88; *Frequency attended* = 81; *Adjusted standardized residual* = 3.9) and fewer cases in participants with at least one item with a score of 2 (*Frequency observed* = 47; *Frequency attended* = 54; *Adjusted standardized residual* = -3.9).

### Discussion

The hypothesis that only a small number of participants with perception-like phenomena could have a psychiatric disorder results confirmed. For a better interpretation of these results, we have to bear in mind that the center where the participants were recruited was not a primary care but a specialized center for ADHD where doctors only visit children and adolescents with more severe symptomatology. Moreover, the results reported concern only the aim of this paper without reporting the presence of dyslexia, enuresis, etc. This could be a limitation, but we postpone a deeper analysis to our next paper.

The significant results can be summarized as follows:

Sixty participants (40%) have at least a score of 2 on the dimension reality/unreality. Thirteen participants of the 60 with at least a score of 2 on the dimension reality/unreality (21.66% of this group and 8.66% of the center participants) exhibited “dissociative symptomatology” in the psychiatric assessment. This dissociative feature always involves the personages of their fantasies that “enter” themselves, a typical dissociative symptom according to DSM 5 criteria. All 13 participants have a very high score on the dimension reality/unreality for this dimension in normal children of lower-secondary, paying attention to the mean age of these children. The phenomenon of the personage that “enters” the subject is very noticeable. This dissociative symptom suggests the possibility that the weak ability to distinguish the unreality of the fantasy attested by the score in this specific dimension of the FFQ could be related to a weakness of the boundaries of consciousness. In dissociative disorder, in fact, the boundaries of consciousness are by definition reduced or abolished, and therefore there is a violation of the first principle previously stated (see “Analysis 2” in the Discussion section of Study 1), with false attribution of thoughts to an external subject (the character who enters into the subject). At the same time, this type of fantasy also violates the second of the principles previously stated, since imaginative thought with the wrong subject also acquires commensurability with sensitive perception, since this character is attributed a real existence.

Moreover, we have the confirmation that probably only the participants with an “extreme” score in the dimension reality/unreality are at risk of a psychiatric disorder, but not the 47 children (78.33%) that have a normal profile.

In two of these adolescents (3.33% of the group of 60) the characters that are present in the visual experiences “entered” in them living as another personality in a persistent way and the diagnosis was Dissociative Identity Disorder, that had developed in a subject previously affected only by ADHD. On the contrary, in the group of 90 children without some significant score in the items of the dimension reality/unreality only two participants were diagnosed as having Dissociative Symptomatology (without ADHD). The difference in the number of participants with Dissociative Symptomatology between the two groups was significant.

None of the children or adolescents who have a weak distinction between reality and imagination had a diagnosis of Psychosis, Schizophrenia or Epilepsy at the visit. In any case we cannot say whether they would develop a major psychiatric disorder after 1–2 years. Finally, we can state that 78.33% of the participants of the ADHD center sample with a perception-like experience have no major psychiatric or neurologic disorder. This datum confirms that some kinds of hallucination are not necessarily pathological (Aziz, 2009). Moreover, it suggests that confusion between reality and unreality could suggest the existence of a special kind of imagination for normal subjects. The psychophysiology of this dimension regards the boundaries between the external world and the her/himself, so its extreme expression could be a disorder of this skill and not a Psychosis as a hallucination might apparently suggest. To our knowledge this is not reported in the previous literature and represents an important novelty to study young people at risk.

The results study 2 might be conditioned by the disorder diagnosed in the participants (ADHD) and the percentage of dissociative symptoms found could be lower in a normal population. Moreover, many ADHD participants have comorbid disorders such as learning disabilities, and some results might be biased. This group is composed of a high percentage of males due to the type of disorder they have, and the age of the participants is not well distributed along the age span 8 to 18. Finally, the lack of use of psychoactive drugs was not verified with a biological assessment (Urine and Blood tests) in the normal and ADHD sample but the exclusion of such bias is only anamnestic.

## General Discussion

All hypotheses expressed in the introduction of this paper were confirmed by the results of both studies. There is substantial agreement between our results and those from past research regarding imaginary playmates and paracosmos, and in addition some new information about the Imaginary relatives. This agreement is an indirect confirmation of the reliability of the remaining data (in addition to the validation results) concerning the five parameters studied. We refer overall to the qualitative analysis of the items concerning reality/unreality and the quantitative results of this dimension. A question remains open: the difficult interpretation of these data needs above all, in our opinion, to better define what it is possible to understand as development of the “boundaries” of consciousness. Some suggestions in this direction are made by the results of neuroimaging and pharmacology research. Sherman et al. (2014) found that the development of the CNS during early adolescence seems to go in the direction of a progressive segregation of the networks and not in the direction of their major extension or “opening.” However, the opposite occurs when a drug with a special and intensive affinity for 5HT2a receptors is administered (Rolland et al., 2014), such as the endogenous hallucinogen dimethyltryptamine (DMT). DMT at high doses produces a loss of boundaries of consciousness, with perception-like phenomena including hallucinations (Strassman et al., 1994a,b). Sampedro et al. (2017) found that DMT seems to increase connectivity, resulting temporarily in a level of connectivity found in a previous stage of development. Probably this return to brain connectivity has the consequence of a destructuring of boundaries between imagination and the external world previously acquired. Moreover, DMT is hypothesized to be responsible for a REM-sleep state in normal subjects. This hypothesis is based on the strong similarity between hallucinogenic DMT effects and dreams, especially lucid dreams (Kraehenmann, 2017). DSM 5 (American Psychiatric Association [APA], 2013, pp. 87–88) does not consider the hallucinations that occur at the beginning of sleeping and at awakening pathological. These hallucinations are very common and also typical of narcolepsy (Leu-Semenescu et al., 2011) and sleep paralysis (O’Hanlon et al., 2011), conditions that can also occur in developmental age.

Typically, hallucinations such as false awakening (a condition in which the subject thinks to be awake but he is sleeping; he realizes that awakening was false when he really wakes up) or visions about personages occur in these disorders in REM sleep with a lucid dream descriptive correlate, and these data are well attested (Fraigne et al., 2014). Therefore, there is the possibility that a weak ability to distinguish one’s own imaginative production from the external world could intersect with the non-pathological hallucinations of these conditions. These data seem to provide a picture of what happens biologically that underlies some types of fantasy and seems to suggest a possible and realistic psychophysiology to the observed phenomena. These suggestions indeed go toward a possible interpretation of non-pathological perception-like experiences as non-perceived and awakening lucid dream condition. At first glance, this hypothesis could appear non-realistic because we are prone to seeing a strong difference between dreams and imagination. Nevertheless, when imagination escapes from the control of consciousness, as we have seen above about the dimension reality/unreality, a common feature with dreams appears and the difference becomes less marked. Moreover, hypnagogic and hypnopompic hallucinations are frequently accompanied by a dissociative feature and meetings with invisible personages in adults (Denis and Puerlr, 2017), in adolescents (Donfrancesco et al., 2017) and in children (Babiker and Prasad, 2015). In these special conditions a regression in the definition of the boundaries of consciousness probably happens. We do not know exactly how the maturation of consciousness and its boundaries happens. Probably in its complex development a progressive augmentation of the control of consciousness over fantasy has a part. Our hypothesis considers, therefore, the possibility that a “bridge condition” could exist between dreams and imagination that we call “Dreamtime.” “Dreamtime” is less pathologising than APS, and its definition could be: “a special type of imagination that shares some features with dreams, and in which the difference between reality and unreality is weak.”

The study of fantasy is very important because imagination represents a pivotal ability in the mental functioning of typically developing children. Our research showed that the study of fantasy may improve the comprehension of the development of consciousness, with special regard to its boundaries.

The quality and quantity of fantasy decreases with age from 8 to 18. Across these ages, imaginary playmates, Imaginary relatives and “Paracosmos” are present. A large number of children and adolescents declare having, sometimes, a type of imagination with a weak ability to distinguish reality from fantasy and so very similar to a perception-like event. This is probably due to a fantasy very rich in quality with low conscious control. The theoretical implication of these findings is that a major frequency of perception-like phenomena is probably due to a slower development of boundaries between her/his own thinking and the external world. Only a few participants with this kind of experience are at risk from dissociative personality identity disorder, generally those with a very frequent perception-like experience. The majority of participants with perception-like experiences are free from serious psychiatric or neurological illness. Therefore, the technical acronym APS could be substituted by the less pathologising word “Dreamtime.” The practical application of the findings of this paper is the possibility of using a questionnaire for a quantitative and qualitative study of fantasy and “Dreamtime” in developmental studies and in the assessment of pathological risk.

## Data Availability

The datasets generated for this study are available on request to the corresponding author.

## Ethics Statement

This observational study was carried out in accordance with recommendations of the Declaration of Helsinki. We collected written informed consent from all subjects.

## Author Contributions

RD realized the questionnaire, designed the study and wrote the manuscript. CV did the statistical analysis and wrote the results section. GP, LB, and MM participated in the writing of the manuscript. AD, PG, EA, FD, AR, and RT collected the data and participated in the writing of several discussion sections. MD designed the study and wrote the manuscript.

## Conflict of Interest Statement

The authors declare that the research was conducted in the absence of any commercial or financial relationships that could be construed as a potential conflict of interest.
