# Supplementary material for: The Validation of the Free Fantasy Questionnaire for Children and Adolescents: From Imaginary Playmate to “Dreamtime”
Source: Front Psychol. 2019 Jun 7;10:1343. doi: 10.3389/fpsyg.2019.01343 (PMC6567922; doi:10.3389/fpsyg.2019.01343)
Supplement: Supplementary file 1 [file Presentation_1.pdf]

APPENDIX

The items of *Free Fantasy Questionnaire*.

Variable	M	SD	Sk	Ku.
1. My fantasies sometimes contain beautiful colours (*)	2.25	1.76	0.23	2.23
2. I spend more than an hour daily fantasising (*)	1.59	1.69	0.81	3.03
3. I sometimes feel as if I'm living in a dream (*)	1.43	1.59	0.94	5.24
4. I'm not sure whether I have fantasised about something or dreamt it (*)	1.40	1.57	0.91	9.59
5. I sometimes speak to a person who doesn't exist in real life but whom I invent using my imagination (*)	0.80	1.44	1.83	7.75
6. I sometimes speak to a person who doesn't exist in real life but whom I invent using my imagination and who has a name, a background and a world he/she lives in (*)	0.68	1.37	2.04	10.35
7. I sometimes think other people are my parents (*)	0.53	1.12	2.40	-0.79
8. I sometimes think other people are my parents and that I know their names and background (*)	0.36	0.98	3.11	2.48
9. I sometimes think I belong to another family (*)	0.41	1.01	2.81	-0.99
10. I sometimes think I belong to another family about whom I know everything (*)	0.36	0.99	3.23	2.38
11. When I fantasise, I choose people I know as the characters for my story	1.59	1.72	0.74	0.05
12. I sometimes think that my real home is not the one I live in but that it is somewhere else (*)	0.75	1.36	1.87	-0.86
13. I sometimes make up very long, beautiful stories (*)	1.78	1.78	0.62	4.01
14. I sometimes make up very long, beautiful stories but that don't seem to be my own, as if it wasn't me who made them up but someone else, who then puts them in my head (*)	0.76	1.33	1.83	-0.72
15. When I fantasise, I enter another world that is very different from my everyday life but that I made up myself (*)	1.24	1.64	1.16	3.60
16. When I fantasize, the images I see are full of details (*)	1.71	1.72	0.66	-1.36
17. I sometimes speak to an invisible character that really exists and that only I can call by doing something (for example, by saying certain words that only I know, by doing particular things that I know he/he likes, etc.) (*)	0.61	1.29	2.23	3.30
18. When I fantasise, I feel very strong emotions (*)	1.63	1.69	0.74	7.68
19. I sometimes speak to a character that is invisible but really exists whom I can ask for help to change something I don't like (this character isn't God or a character that belongs to your religion) (*)	0.62	1.25	2.12	4.69
20. I have control over my fantasy and can do whatever I like with it (*)	2.03	1.92	0.40	6.06
21. There are some fantasies that seem to have entered my head from another person and over which I don't have control, which means they go on by themselves. (*)	0.67	1.27	2.03	9.40
22. I sometimes speak to a character that is invisible to other people but that really exists and that orders me to do things (*)	0.42	1.09	2.87	0.66
23. I sometimes speak to a character that is invisible to other people but that really exists and that gives me a lot of explanations for what happens (*)	0.55	1.20	2.34	0.87
24. If I'm not sure what to do, I call a character that is invisible to other people but that really exists and that advises me on what to say and do. (*)	0.48	1.12	2.58	2.17
25. I sometimes speak with an Alien that is invisible to other people but that I can see, and that is real and does exist (*)	0.37	1.06	3.16	-0.80
26. When I fantasise, my mind wanders to a city that is not the one I live in and that has rules that are not those we have in this world (*)	1.07	1.52	1.35	4.63
27. When I fantasize, I sometimes end up in an invisible, secret place where other people can't follow me (*)	0.98	1.55	1.46	-1.19
28. When I fantasise, I find myself speaking to characters that I have not invented but that somehow enter my fantasy (*)	0.78	1.37	1.79	2.23
29. When I fantasise, I feel as if I'm in a film (*)	1.63	1.71	0.71	3.03
30. I sometimes speak to invisible characters from an invisible world that really exists but that not everyone can see, and these characters turn up on their own without me having ever called them (*)	0.56	1.22	2.34	5.24
31. When I fantasise, I'm the one who makes up all the characters (*)	1.81	1.92	0.59	9.59

Note. (*) = the item distribution resulted significantly different than a Gaussian curve.
